# Second primary breast cancer after Hodgkin's disease

**DOI:** 10.1038/sj.bjc.6601499

**Published:** 2004-01-20

**Authors:** A Horwich, A J Swerdlow

**Affiliations:** 1 Academic Unit of Radiotherapy and Oncology, The Royal Marsden NHS Trust and the Institute of Cancer Research, Downs Road, Sutton, Surrey SM2 5NG, UK; 2 Aetiological Epidemiology, The Royal Marsden NHS Trust and the Institute of Cancer Research, Downs Road, Sutton, Surrey SM2 5NG, UK

## Abstract

Although the potential carcinogenic risk of radiotherapy is well known, it has become clear that there is a particularly high risk of radiation-induced breast cancer in women treated for Hodgkin's disease at young ages. Thankfully, death from breast cancer in this population is uncommon, but it is important to understand factors contributing to the risk, including treatment parameters, and to develop a logical and efficient method for medical management of those at risk. In this minireview, we examine the evidence which should inform such a management policy.

The prognosis of patients with Hodgkin's disease has been improved dramatically over the last 40 years by the development of extended field radiotherapy techniques and then by combination chemotherapy (DeVita *et al*, 1980; Rosenberg and Kaplan, 1985). However, long-term follow-up of survivors has demonstrated that a price of this success has been an increased risk of second cancers. Acute leukaemia frequently occurs in the first decade after treatment, mainly as a consequence of chemotherapy regimens that included an alkylating agent (Tucker *et al*, 1988; Kaldor *et al*, 1990; Swerdlow *et al*, 2000). In the long term, however, the absolute excess risks of a second solid cancer are higher (Swerdlow *et al*, 2000; Ng *et al*, 2002b; van Leeuwen *et al*, 2003) and are linked mainly with radiotherapy, although for some sites, such as lung cancer, there may be a substantial risk also from chemotherapy (Swerdlow *et al*, 2000; Travis *et al*, 2002). Recent reports from The Netherlands (van Leeuwen *et al*, 2003) and from an international consortium of cancer registries (Travis *et al*, 2003) have refined our understanding of how therapy may affect breast cancer risk.

The relative risks of many cancers are higher if patients have been treated for Hodgkin's disease at a younger age (Swerdlow *et al*, 2000; van Leeuwen *et al*, 2000; Dores *et al*, 2002). Thus, although the risk of breast cancer is only about 50% increased in most all-age studies of women with Hodgkin's disease (van Leeuwen *et al*, 1999), relative risks are far larger than this in patients treated at young ages, especially those treated in childhood and adolescence (Hancock *et al*, 1993; Bhatia *et al*, 1996b); for them this is one of the main long-term sequelae of radiotherapy. This effect of age is unsurprising, as similar gradients are seen in other cohorts of radiation-exposed women (United Nations Scientific Committee on the Effects of Atomic Radiation, 1994).

The size of risk of breast cancer found in Hodgkin's disease patients has varied between studies. For patients treated under age 21 years relative risks have generally been around 15–25 (Hancock *et al*, 1993; Sankila *et al*, 1996; Wolden *et al*, 1998; Metayer *et al*, 2000; van Leeuwen *et al*, 2000; Dores *et al*, 2002), although they have been less raised (Neglia *et al*, 2001) or more raised (Mauch *et al*, 1996; Bhatia *et al*, 1996b; Aisenberg *et al*, 1997; Ng *et al*, 2002b) than this in a few studies; absolute excess risks have generally been of the order of 20–40 per 10 000 per annum. Relative risks have been greater for patients treated at ages 10–16 than at younger or older ages (Hancock *et al*, 1993; Sankila *et al*, 1996; Bhatia *et al*, 1996b; Metayer *et al*, 2000). Differences between study results partly reflect differences in distributions of age at treatment, radiation dosage and duration of follow-up, but also caution must be exercised in interpretation of studies such as that by Bhatia (Bhatia *et al*, 1996b), where follow-up to a fixed end-date is incomplete. Such incompleteness will tend to be predominantly for subjects who have not had a second malignancy and hence will artefactually increase apparent risks in relation to years of successful follow-up (Donaldson and Hancock, 1996). Similarly, estimates of cumulative risk of breast cancer after treatment at young ages have at the extreme been estimated at 42% at 30 years of follow-up for women treated under age 16 years in a study with incomplete follow-up (Bhatia *et al*, 1996a), but studies with high-quality follow-up have found cumulative risks of 12% at 30 years after treatment in the Nordic countries (Sankila *et al*, 1996) and 16% at 25 years in the Netherlands (van Leeuwen *et al*, 2000) for patients treated under age 21 years. In median 18-year follow-up of over 500 women treated at ages under 41 years in the Dutch cohort, only five deaths occurred from breast cancer (Aleman *et al*, 2003), and among 161 deaths in long-term follow-up of 494 women treated under age 51 years in the US, four were from breast cancer (Ng *et al*, 2002a)

Risks of second primary breast cancer are also raised after treatment for Hodgkin's disease in young adulthood, although relative risks are lower than those for childhood and adolescent treatment (Hancock *et al*, 1993; van Leeuwen *et al*, 2000; Ng *et al*, 2002b), reflecting the greater background rates at older ages. However, a lesser relative risk does not necessarily imply a lesser absolute excess, and indeed van Leeuwen *et al* (2000) in the Netherlands and Hancock *et al* (1993)and Ng *et al* (2002b) in the US found absolute excess risks fairly similar for patients treated at ages 20–29 years to those for patients treated under age 20 years. For treatment at ages 30 years and above, most studies have found no raised risk (Hancock *et al*, 1993; Aisenberg *et al*, 1997; Swerdlow *et al*, 2000), although in a large Dutch cohort there was a relative risk of 2.4 (0.9–5.2) for patients treated at ages 31–39 years (van Leeuwen *et al*, 2000) and in a US cohort there were relative risks of 3.7 (1.0–9.5) for women treated at 30–35 years and 3.4 (0.4–12.1) for those treated at 36–40 years) (Ng *et al*, 2002b) It thus seems possible that the raised risks extend a little beyond age 30 years, but it is unclear how far.

The cause of the raised risk of breast cancer patients treated for Hodgkin's disease is exposure to radiotherapy fields that involve the breast. Most of the data discussed above, however, relate to women treated for Hodgkin's disease overall, and thus risks are greater if only patients exposed to radiotherapy are considered. For instance, in a UK cohort (Swerdlow *et al*, 2000) the relative risk of breast cancer for women treated under age 25 years was 7.7, but for those treated by radiotherapy alone at these ages it was 14.4.

In adults, at least, the induction period before breast cancer is observed is long, with significantly raised risks appearing only at 10–14 (Swerdlow *et al*, 2000; Dores *et al*, 2002; Ng *et al*, 2002b) or 15–19 (Hancock *et al*, 1993; van Leeuwen *et al*, 2000) years after first treatment. After childhood or adolescent treatment, however, induction periods appear to be shorter (Bhatia *et al*, 1996b; Sankila *et al*, 1996; Travis *et al*, 1996; Metayer *et al*, 2000), indeed as short as within the first 5 years in one study (Bhatia *et al*, 1996b).

As a consequence of the long induction periods in adults, the great majority of breast cancers after Hodgkin's disease radiotherapy occur after age 30 years. How long the risks remain raised is as yet unknown, because of the limited length of follow-up yet available in published studies. Several groups have presented data for 20 and more years of follow-up, finding large and significant relative risks of breast cancer for this time period (Hancock *et al*, 1993; Bhatia *et al*, 1996b; Wolden *et al*, 1998; Metayer *et al*, 2000; van Leeuwen *et al*, 2000). One study has published cohort results for 25 and more years, again with a significantly raised risk (Dores *et al*, 2002) (in a case–control study with no general population comparison, the risk was nonsignificantly raised at 25 and more years for radiation-exposed women (Travis *et al*, 2003)). Relative risks in these studies have usually been somewhat diminished in the longest follow-up period compared with the period preceding it, whereas absolute excess risks, when these have been published, have been at least as great as in the preceding period (Hancock *et al*, 1993; Wolden *et al*, 1998; van Leeuwen *et al*, 2000). Although one cannot be sure what will occur at yet longer follow-up periods, judging from other radiation exposed groups it would be expected that there would be substantial raised risks persisting through 40 or more years of follow-up (Committee on the Biological Effects of Ionizing Radiation (BEIR), 1990).

Breast cancers occurring after treatment for Hodgkin's disease are more likely to be bilateral than breast cancers in general (Yahalom *et al*, 1992; Basu *et al*, 2002). There is little information on the molecular phenotype of breast cancers after Hodgkin's disease; in one study 10 out of 18 tumours examined were oestrogen receptor negative (Yahalom *et al*, 1992). A study of 19 tumours found no apparent difference between post-Hodgkin's and sporadic breast cancers in loss of heterozygosity or in TP53 and K-*ras* mutations, and a nonsignificantly raised frequency of micosatellite alterations in the post-Hodgkin's disease tumours (Behrens *et al*, 2000). Two studies have described raised risk of breast cancer in Hodgkin's disease patients who have undergone splenectomy or splenic irradiation (Dietrich *et al*, 1994; Bhatia *et al*, 1996b), but there are insufficient data to regard this as an established association. Based on limited data, no clear relation has been found with reproductive history (Travis *et al*, 2003).

It would be of great interest to know whether radiotherapy-related risks interact with those from genes affecting breast cancer susceptibility, but there is almost no information on this. Bhatia *et al* (1997) found no excess of breast cancer in relatives of patients with breast cancer after Hodgkin's disease, but this was based on small numbers. Because carriers of the ataxia telangiectasia (AT) gene have increased *in vitro* sensitivity to radiation, and have been found to have raised risk of breast cancer (Ramsey *et al*, 1996), they might be a group for whom radiotherapy constitutes a particular hazard. Genetic analyses of 32 patients developing breast cancer more than 10 years after radiotherapy for Hodgkin's disease did not reveal evidence of truncating mutations (Broeks *et al*, 2000). In a study based on AT gene sequencing which compared HD patients with or without second breast cancers, risk of breast cancer was not associated with either protein-truncating or with excess missense mutations (Offit *et al*, 2002).

Because much of our knowledge of risk of breast cancer after Hodgkin's disease is derived from treatments carried out in the 1960–1980s, there is relatively little information about the likely impact of more recent radiotherapy practices, such as involved field radiotherapy and reduced-dose radiation. Involved field radiation is often employed following chemotherapy in early stage Hodgkin's disease and is confined to overtly involved lymph nodes plus a margin along the node chain. Clearly for presentations confined to cervical lymph glands, breast irradiation would be minimal, and in practice the major component of breast irradiation derives from treating the axilla, which is a relatively uncommon site for early Hodgkin's disease. However, mediastinal involvement is not uncommon and fields to the central chest will necessarily irradiate medial breast segments. A nested case–control study from the Netherlands (van Leeuwen *et al*, 2003) included an estimate from radiotherapy charts as well as dosimetric measurements and planning computer simulations of radiation doses to the local area of the breast where the tumour had developed. The risk of breast cancer increased highly significantly with increasing radiation dose (*P* trend=0.002), with a relative risk of 4.5 (1.3–16) for ⩾38.5 Gy compared with <4 Gy. This was also shown in the international case–control study (Travis *et al*, 2003) (*P* trend <0.001), which found a RR of 8 in those treated to a dose of more than 40 Gy to the breast, compared with less than 4 Gy. The few other studies that have investigated the relation of risk to dosage have not calculated doses specifically to the part of the breast in which the cancer occurred. Two studies found evidence of a dose–response relation (Bhatia *et al*, 1996b; Tinger *et al*, 1997) while one did not (Hancock *et al*, 1993), although the great majority of person-years in the latter study were in one dose category. There are inaccuracies in retrospective dose allocations in such studies because radiotherapy causes breast shrinkage, leading to changes in breast shape of treated women over time. Nevertheless, the data from van Leeuwen's study are strong, and they accord with what one might expect from other epidemiology, so it appears likely that reduction in dosage will reduce the risk of breast cancer.

Chemotherapy alone does not appear to be associated with an increased risk of breast cancer after Hodgkin's disease (Swerdlow *et al*, 2000; van Leeuwen *et al*, 2000; Dores *et al*, 2002), and indeed the available data would be compatible with a reduced risk. Analyses of patients treated with both chemotherapy and radiotherapy have in several studies (Gervais-Fagnou *et al*, 1999; Swerdlow *et al*, 2000; van Leeuwen *et al*, 2000, 2003; Travis *et al*, 2003) but not all (Hancock *et al*, 1993; Aisenberg *et al*, 1997) suggested a reduced risk compared with that after radiotherapy alone. In the British cohort, the relative risk for patients treated at less than 25 years of age was 14.4 after radiotherapy alone and 4.6 after radiotherapy plus chemotherapy (Swerdlow *et al*, 2000). In the Dutch cohort a similar reduction was seen for patients receiving mixed therapy (van Leeuwen *et al*, 2003), and indeed the effect of radiation dose on breast cancer risk was only seen in patients who had not received chemotherapy. The diminished risk after chemotherapy was not due to radiation dose reductions in these patients (van Leeuwen *et al*, 2003). Although the diminished risk could in principle be due to a direct effect on initiated breast epithelial cells, it seems most likely that it is due to ovarian toxicity and consequent hormonal ablation. A nested case–control study from the Netherlands (van Leeuwen *et al*, 2003) found strong evidence favouring hormonal ablation: 48 case patients who developed breast cancer 5 or more years after treatment for Hodgkin's disease were compared with 175 matched controls in whom breast cancer had not occurred. In total, 69% of controls who had been treated with more than six cycles of chemotherapy plus radiotherapy reached the menopause before the age of 41 years *vs* only 9% of controls treated with radiotherapy alone. Reaching menopause before age 36 years was associated with a greatly reduced risk of breast cancer (RR 0.06, 95% CI 0.01–0.45) and risk increased steeply with number of premenopausal years after treatment: relative risks were 0.15, 0.24 and 0.91 for <5, 5–14, and ⩾15 premenopausal years, respectively. Travis *et al* (2003) found that breast cancer risk decreased significantly (*P*=0.003) with increasing number of alkylating agent cycles and decreased, although the trend was not significant, with younger age at menopause. Thus ovarian hormones may be critical to carcinogenesis after radiation initiation. Such an effect would give a potential explanation of why breast cancer risk decreases with older age at irradiation, as there will be fewer or no years before menopause in patients who are older at radiotherapy. It is notable that after radiotherapy to the ovaries for either HD (Travis *et al*, 2003) or other conditions (Boice *et al*, 1985; Darby *et al*, 1994)., there is also a reduction in risk of breast cancer, presumably mainly due to ovarian failure, although some risk reduction has been found even at postmenopausal ages (Boice *et al*, 1985; Darby *et al*, 1994). For Hodgkin's disease, the relation to chemotherapy-induced menopause needs further investigation. Analyses of risk in patients treated in the more recent past with chemotherapy combinations that do not cause ovarian failure, such as adriamycin, bleomycin, vinblastine and dacarbazine (ABVD) (Santoro *et al*, 1982), may help to clarify this issue.

What should be the clinical response to this risk, given the admittedly incomplete data available? Breast cancer is a common disease with more than 39 000 cases per year diagnosed in the UK; the incidence is 45 per 100 000 women per annum for women aged in their 30s rising to 300 per 100 000 in those in their 80s. Thus, even a modest increase in relative risk is of considerable concern. Issues to consider include advising and counselling patients at risk, the possible benefit of screening or prevention programmes, the impact on current treatment practices for Hodgkin's disease, and the research needed to enable judgement and minimisation of risk in the future. In the UK, the Department of Health is taking steps to ensure appropriate counselling of women at risk. From existing databases, we estimate that there are approximately 2600 women alive and at risk in Britain, although many of these may already have been informed about their risk by physicians in their follow-up clinics. It is important that the risk level be explained in a readily comprehensible way and for most patients this might be in terms of the expected excess number of breast cancers for every 100 years of follow-up as judged from individual risk factors. More detailed risk data than currently available are needed, however, specified by duration of follow-up, age at treatment, and dose, to enable fully personalised advice.

There is evidence that regular mammographic screening may reduce breast cancer mortality. However, this is derived from screening predominantly women more than 50 years of age. In younger women X-ray mammography may be less sensitive because many have increased density of breast tissue, although there is growing evidence that there is some beneficial effect (Tabar *et al*, 2003). Magnetic resonance imaging is being evaluated to screen younger women and evidence to date suggests that it is a sensitive technique but there may be a higher false-positive rate (Warren and Crawley, 2002). X-ray mammography involves less than 1/1000th of the radiation dose used in treatment of Hodgkin's disease and can be considered for most women at increased risk. In a US study in which Hodgkin's disease patients underwent mammography annually (or every second year before age 30 years), all 12 breast cancers occurring during follow-up were evident on mammogram (Diller *et al*, 2002) and in another, retrospective study, 26 out of 29 cancers were demonstrated on mammography (Dershaw *et al*, 1992). Because screening needs to start earlier than clinical cancer risk is raised if it is to serve the purpose of early detection, it would seem reasonable that for women who were given radiotherapy at ages 20–30 years screening should begin at about 8–10 years postradiotherapy. For women treated before age 20 years, it may be reasonable to add that screening should not start before a fixed attained age – say about 25 years – because of the rarity of second primary breast cancers before ages in the late 20s (Aisenberg *et al*, 1997). As to frequency of screening, it may be reasonable to follow the procedure for women at high genetic risk of breast cancer, and (in the UK) to recommend annual screening up to age 50 years when the national programme begins, and then to add an additional screening between the three yearly national programme appointments in the light of higher risk and desirability to limit the rate of interval cancers.

With regard to continued use of hormone replacement therapy (HRT) in patients with premature menopause, or hormone-based chemopreventative strategies such as Tamoxifen for those at risk, there are insufficient data to give clear advice. In the study by van Leeuwen (van Leeuwen *et al*, 2003), risk was not significantly increased in users of HRT, but the number of cases who had used HRT for more than a few years was small and the confidence interval was wide. The information on the possible protective effect of chemotherapy discussed above suggests that hormone ablation may reduce cancer risk, but against this must be weighed the health benefits of HRT, for example in combating osteoporosis. There is a case to consider a chemoprevention trial in patients at risk of breast cancer, although patient willingness to enter such a trial would need to be evaluated carefully to ensure there were sufficient numbers to demonstrate whether there is an effect.

With regard to treatment of future patients, there is a need to take account of the breast cancer risks that would arise from radiotherapy, but to note that these apply only to a subset of all Hodgkin's disease patients (young women), not to all, and that a balanced view of all the benefits and potential long-term adverse effects needs to be taken when selecting treatment. There is a trend in current treatment of Hodgkin's disease toward reduced use of radiotherapy. This comes from lack of clear evidence for overall survival benefit in advanced and disseminated disease presentations (Specht *et al*, 1998). In early disease, combined modality protocols are used in which chemotherapy is followed by involved field radiation and doses of radiotherapy are reduced (e.g. Sieber *et al*, 2000). There are limited data on chemotherapy alone in early Hodgkin's disease, and it is uncertain whether disease control rates are as high as with radiotherapy or combined modality approaches (Biti *et al*, 1992). A current Canadian trial is investigating ABVD in this role. Furthermore, the advent of more sensitive response evaluation based on positron emission tomography (Spaepen *et al*, 2001) may allow judgement of which patients with early Hodgkin's disease may completely avoid radiotherapy after initial response to chemotherapy.

For those women who do require supradiaphragmatic radiotherapy at ages less than 30 years, it is important to seek to minimise dose and limit volume of breast tissue in the field and to ensure that they are informed of, and accept, the associated risk of second primary breast cancer.
